# 

**Published:** 1851-11

**Authors:** 


*Medical Department of the University of Buffalo.* — The regular session in this institution of 1851 – 2, commences on Wednesday the 5th instant. The courses on Anatomy, by Prof. Palmer; on Surgery, by Prof. Hamilton; on Medicine, by Prof. Flint; on Chemistry, by Prof. Hadley, and on Physiology, by Prof. Dalton, will commence at once, and, with the exception of the anatomical course, will be continued through the term.

The course on Midwifery will, probably, commence soon after the return from Europe of Prof. White, who is expected to arrive in a few days after the beginning of the term.

The course on Materia Medica and Pathology, by Prof. Lee, will commence in January.

The forenoons of Wednesday and Saturday are to be devoted to observations, surgical operations and clinical lectures, at the hospital. On Wednesday afternoons, a medical and surgical *clinique* will be held at the college. Practitioners sending patients to the college for surgical operations or medical advice, are requested to direct their attendance on Wednesday afternoon.

During the preliminary term, which closes at the commencement of the regular term, about fifty students have been in attendance.

---

Several short articles prepared for this No. are excluded for want of space. Communications from Drs. Hunt and Ritter have been received, and will appear in the Dec. No.

